# Estimation of malaria parasite reservoir coverage using reactive case detection and active community fever screening from census data with rapid diagnostic tests in southern Zambia: a re-sampling approach

**DOI:** 10.1186/s12936-017-1962-1

**Published:** 2017-08-07

**Authors:** Joshua Yukich, Adam Bennett, Rudy Yukich, Logan Stuck, Busiku Hamainza, Kafula Silumbe, Tom Smith, Nakul Chitnis, Richard W. Steketee, Timothy Finn, Thomas P. Eisele, John M. Miller

**Affiliations:** 1 Center for Applied Malaria Research and Evaluation (CAMRE), Tulane University School of Public Health and Tropical Medicine, New Orleans, LA 70112 United States; 2 Global Health Group, University of California San Francisco, San Francisco, CA United States; 3 Sensorstar Inc., Ellicott City, MD United States; 4 National Malaria Control Centre, Ministry of Health, Lusaka, Zambia; 5 Malaria Control and Elimination Partnership in Africa (MACEPA), PATH, Lusaka, Zambia; 6 Swiss Tropical and Public Health Institute, and University of Basel, Basel, CH Switzerland

**Keywords:** Malaria, Reactive case detection, Active community fever screening, Resampling, Case detection, Fever screening

## Abstract

**Background and methods:**

In areas where malaria transmission has been suppressed by vector control interventions many malaria control and elimination programmes are actively seeking new interventions to further reduce malaria prevalence, incidence and transmission. Malaria infection prevalence and incidence has been shown to cluster geographically, especially at lower transmission levels, and as such a reactive strategy is frequently used, by which index cases presenting to a passive surveillance system are used to target small areas for testing and treatment, reactive case detection (RCD), or focal drug administration (fDA). This study utilizes geo-located data from a census with parasitological testing with rapid diagnostic tests (RDTs) and treatment-seeking data collection conducted in southern Zambia to estimate the coverage of RCD or fDA in terms of the population and parasite reservoir as well as the operational requirements of such strategies, using a re-sampling algorithm developed exclusively for this purpose. This re-sampling algorithm allows for the specification of several parameters, such that different operational variants of these reactive strategies can be examined, including varying the search radius, screening for fever, or presumptive treatment (fDA).

**Results:**

Results indicate that RCD, fDA and active fever screening followed by RCD, even with search radii over several hundered meters will only yield limited coverage of the RDT positive parasite reservoir during a short period. Long-term use of these strategies may increase this proportion. Reactive strategies detect a higher proportion of the reservoir of infections than random searches, but this effect appears to be greater in areas of low, but not moderate malaria prevalence in southern Zambia.

**Discussion:**

Increases in the sensitivity of RDTs could also affect these results. The number of individuals and households that need to be searched increase rapidly, but approximately linearly with search radius.

**Conclusions:**

Reactive strategies in southern Zambia yield improved identification of the parasite reservoir when targeted to areas with prevalence less than 10%. The operational requirements of delivering reactive strategies routinely are likely to prevent their uptake until prevalence falls far below this level.

## Background

In areas where malaria transmission has been suppressed by vector control interventions many malaria control programmes actively seek new interventions to further reduce malaria prevalence, incidence, and transmission. Additionally, programmes which are considering or have undergone a re-orientation towards malaria elimination may be seeking interventions to actively reduce or eliminate remaining foci of infection. Malaria infection prevalence and incidence have been shown to cluster geographically, especially at lower transmission levels [1–3], and as such a reactive strategy might be utilized by which index cases identified by a passive surveillance system are used to target small areas for malaria testing and treatment/investigation [reactive case detection (RCD)] or focal drug administration (fDA). The principles behind the deployment of these types of reactive strategies derive from similar epidemiological foundations to ring vaccination [4] and might provide efficient ways to target mass drug administration (MDA) or mass testing and treatment (MTAT) interventions to small geographic areas with unusually high infection or transmission rates.

Little is known, however, about the effectiveness of the use of reactive approaches to guide MDA and MTAT, and few studies have made an attempt to estimate the potential of reactive strategies to cover high proportions of the reservoir of infections [5–7]. A number of studies have summarized the prevalence of malaria amongst household contacts of passively detected index cases, however, these studies did not include an appropriate comparison group, thus the gain in reservoir coverage of RCD, or related approaches, in those settings could not be ascertained [2, 3, 8–10]. While malaria is known to cluster geographically, the level and nature of clustering may vary with prevalence, population malaria exposure history, geographic features of the landscape, the built environment, human settlement patterns and various other factors [11].

In order to effectively plan for the deployment and testing of RCD or fDA strategies, when and if they are used, it will be necessary for malaria control programmes to make local assessments of both the expected coverage of the intervention in operational and parasitological terms as well as the resource requirements and the ideal search strategy to use in the response to index case identification.

In Zambia, malaria vector control, intermittent preventive treatment in pregnancy (IPTp) and treatment with artemisinin-based combination therapy (ACT) have been scaled up nationwide. These interventions, along with community case management, have reached high and sustained coverage, resulting in significant reductions in the malaria burden in some areas [12, 13]. In the wake of these successes the Zambian National Malaria Control Centre (NMCC) and partners are in the process of testing new strategies to further reduce the malaria burden with the ultimate goal of malaria elimination and the shorter term goal of creating malaria free areas within the country. These strategies include the expansion of community health worker (CHW) led case management and CHW led RCD. In order to rationally plan and select interventions to achieve these goals, operational, effectiveness and cost-effectiveness information about these relatively un derstudied reactive strategies is urgently needed.

This study utilizes geo-located data from a census of 23 health facility catchment areas with parasitological testing and treatment-seeking data collection, which was conducted in southern Zambia. These data were used to estimate the coverage of RCD or fDA in terms of the total population and the parasite reservoir as well as the operational requirements of such strategies, using a re-sampling algorithm developed exclusively for this purpose.

## Methods

### Study site

**Fig. 1:**
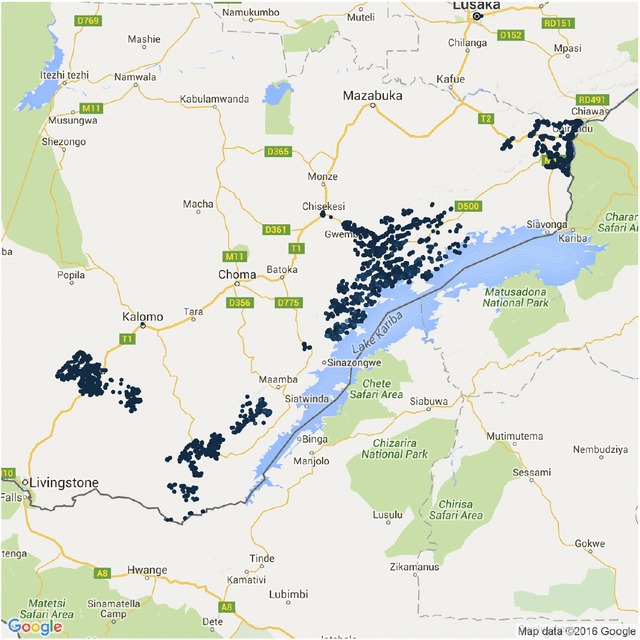
Study area map. Map of study area showing households

Southern Province, Zambia is an area of moderate but heterogeneous malaria transmission. In the years 2012 and 2013, six rounds of dry-season MTAT were conducted in four districts as part of a community randomized controlled trial. The current study utilizes data from one round of data collection covering 23 health facility catchment areas. The study site has been described in detail elsewhere [14]. Figure 1 is a map of the study area.

### Data and data collection

During each MTAT round, CHWs systematically went door to door and screened all individuals in their target areas using Ministry of Health (MoH) approved RDTs (*SD Bioline Pf* and *ICT Mal Pf* brands); both detect histidine-rich protein 2 (HRP2). CHWs conducted the screening alongside survey teams collecting household and individual level data. Data were entered using personal digital assistants equipped with GPS devices. The data collected among consented households and individuals included household geo-location, household composition, RDT positivity/negativity, household bed net ownership, individual fever history and treatment seeking for malaria among all household residents. Data collection has been described in more detail previously [14].

### Definitions

- **Search criteria:** The parameters of an active or reactive strategy which can be varied in the re-sampling algorithm. The search criteria are listed below.
  - **Coverage of household search:** The probability that a household which should be searched according to the presence of an index case and meeting search criteria is actually searched.
  - **Individual treatment-seeking probability:** An individual’s probability of seeking care given that they experienced febrile symptoms. Treatment-seeking was also observed in the census dataset and in some re-sampling experiments is used directly as observed.
  - **Search radius:** If a distance based search is used, this is the distance from the index case household within which all other households will be targeted for a search.
  - **Number of nearest households:** If a nearest neighbour search type is selected, this is the number of the nearest households which will be searched.
  - **Sensitivity and specificity of tests:** The sensitivity and specificity of the diagnostics used to identify malaria parasite infections. These can be specified separately for index case identification and for follow up at the household during the search.
  - **Coverage of individuals:** The probability that an individual in a searched household is actually tested or treated.
- **Parasitological or parasite reservoir coverage:** The proportion of all parasite positive individuals estimated to be treated in a search.
- **Operational coverage:** The proportion of all individuals in the population contacted during a search.
- **Resource requirements of a search:** The number of individuals or households that are targeted for follow up in a search. This is a proxy for the cost of a search.

### Re-sampling algorithms

**Fig. 2:**
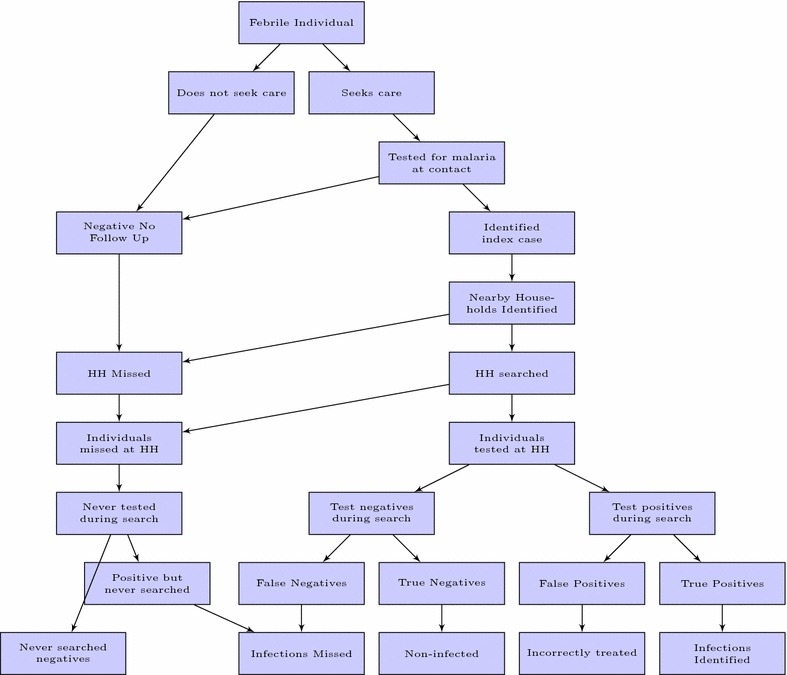
Schematic diagram of the reactive case detection re-sampling algorithm. *Lowest level* shows classification of individuals by measured infection status and identification by the system. False positives and false negatives are those whose parasite status in the original census data were expected to be misidentified by the reactive system

A re-sampling algorithm was developed using the [R] programming language [15]. The basic algorithm follows the following steps and is shown schematically in Fig. 2:

1. Identify potential index cases from the data. Fever and/or treatment seeking history is used to select potential index cases.
2. Identify actual index cases based on the conditional probability of testing positive at a health facility or during an active fever case detection sweep.
3. Use index cases to identify potentially searched households and individuals in the community given a set of search criteria.
4. Determine the number of infected individuals identified in the search and record the number of houses searched, individuals tested and estimate the proportion of the reservoir identified.
5. Store these results as outputs of the iteration.
6. Repeat the process for a pre-specified number of re-samples in order to establish mean performance and credibility bounds for each chosen parameter combination.

The re-sampling algorithm allowed us to vary several parameters, such that different operational variants of RCD could be examined, including RCD with a distance from index case household approach (e.g. search all households within a 50 m radius for additional cases), or a nearest neighbour based approach (e.g. search teams search the nearest 10 houses), active fever screening (i.e. search teams search the community for individuals with fever or a recent history of fever, then test these individuals for malaria infections and some nearby neighbouring households) or a fDA approach (e.g. individuals are identified in a manner similar to RCD but instead of testing, are treated regardless of parasitemia status). The parameters which can be varied in the algorithm are as follows: (1) the search radius or the number of nearest households searched, (2) the sensitivity and specificity of diagnostics used in the facility or during the resulting household search in the community, (3) the individual treatment-seeking probability, (4) the coverage of the household search, (5) the coverage of individuals within households during the household search, and (6) the sensitivity and specificity of the diagnostic used during the reactive household search (these can be manipulated separately from the initial test applied above).

**Table 1:** Example re-sample results

Outcome	Total	Unique
Index cases	354	NA
Households searched	549	401
Persons tested	284	203
True positives found	155	112
False positives found	1	1
True negatives found	122	89
False negatives found	1	1

The total column presents results based on the assumption that each targeted household and individual are searched multiple times if they are identified by association with multiple index cases, whereas the unique column assumes that houses and individuals can only be searched once in a given search period

Outputs include several summary measures for each simulation (see example output in Table 1): (1) the number of households searched based on the algorithm, (2) the number of households which were searched assuming that a search would only be conducted once per household during the period of time represented by one re-sample, (3) the number of index cases, (4) the total number of infected individuals identified, (5) the total number of negative individuals included in the search, (6) the total number of persons tested/searched/treated, (7) the total number of unique persons tested/searched/treated (e.g. assuming that an individual would only be tested once during the period of time represented by one re-sample).

Additionally the dataset also provided the total number of households in the search area, the total number of persons in the search area including RDT results for all of these individuals. All iterations were conducted at the health facility catchment level [14].

### Analysis

These data allowed for the calculation of parasitological coverage as defined above. The data from each re-sample were summarized, and credibility intervals were calculated by taking appropriate quantiles of the output. Data were analysed either by pooling data across all catchment areas (resulting in a weighted analysis across the entire study area) or by first summarizing within each catchment area and then taking the catchment level estimates as individual data points and summarizing across the entire study area (resulting in an analysis that treated each catchment area as being of equal weight regardless of population size or malaria prevalence).

### Operational analysis

Operational analysis consisted of summarizing outputs including the number of index cases, and the number of houses searched and individuals tested, treated, or identified with parasite infections. These parameters were summarized in terms of means across all simulations with a given set of search criteria and the credible intervals generated by taking appropriate quantiles of the resulting distribution of outputs.

## Results

**Fig. 3:**
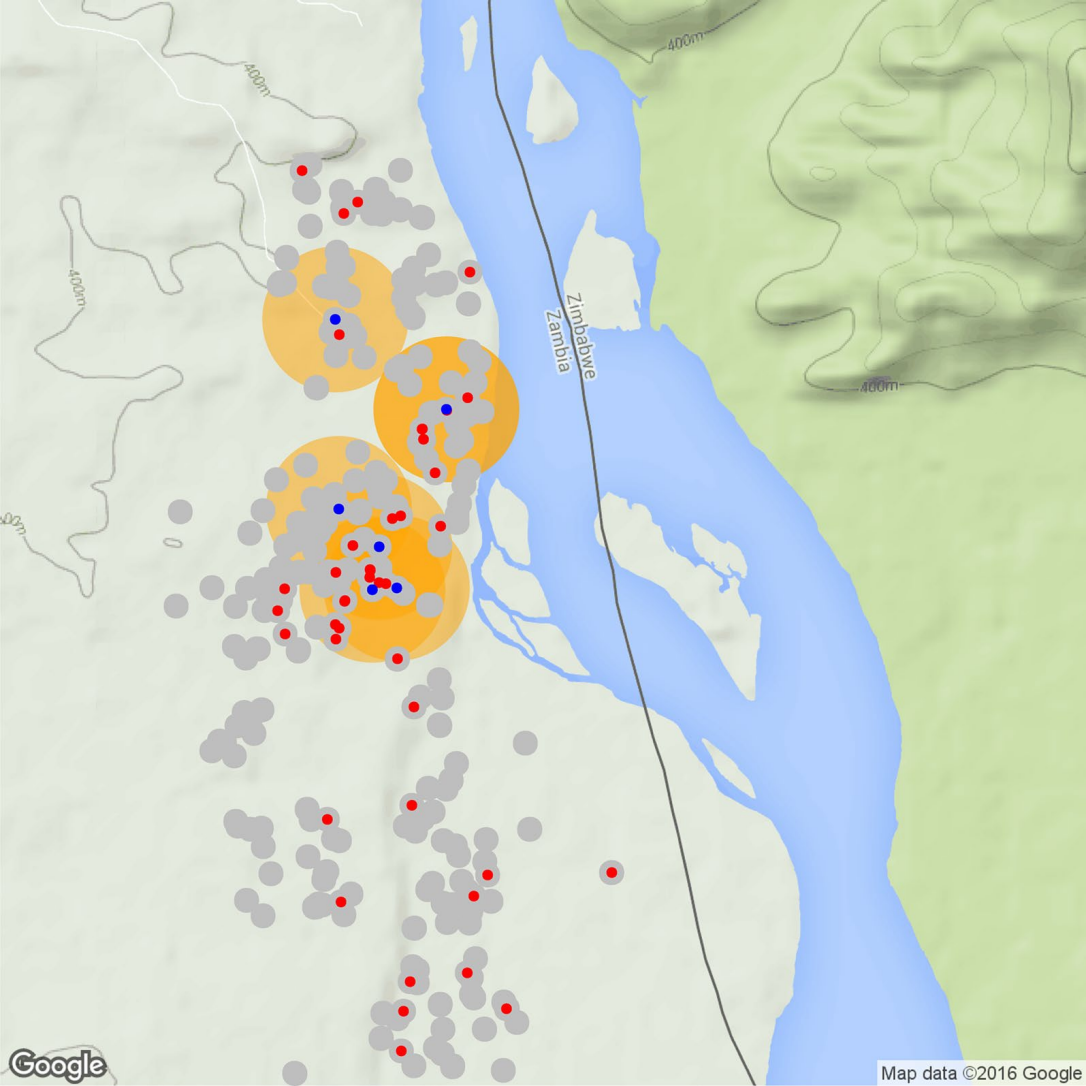
Single simulation schematic result. Index cases shown in *blue*, searched areas shown with *orange circles*, households shown in *gray*, malaria infected individuals shown in *red*

Based on these re-samples, the likely coverage and resource requirements for RCD in the context of southern Zambia was estimated. Figure 3 shows a small scale representation of the results of a single simulation. These results are for the first dry season round only.

### Reactive case detection (sweeping radii or number of households searched)

**Fig. 4:**
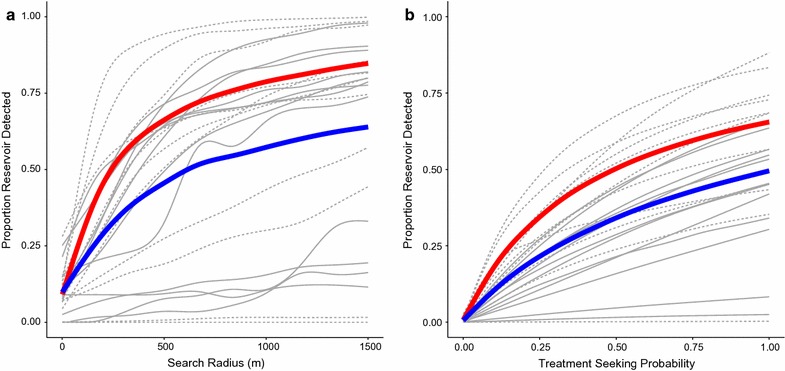
Reservoir detected vs. search radius and treatment seeking probability. **a** Proportion of reservoir detected vs. search radius. Treatment seeking is not simulated and is based on the data. **b** Proportion of reservoir detected vs. treatment seeking probability. Treatment seeking behavior is simulated amongst those reporting fever. *Red line* population aggregate, *solid gray lines* catchment areas with prevalence below the median (92/1000), *dashed gray lines* catchment areas with prevalence above the median (92/1000), *blue line* average over catchment areas. Field test sensitivity = 0.95, field test specificity = 0.80, P (treatment seeker is tested) = 0.90, P (RCD-selected household is covered) = 0.90, P (individual within RCD-selected household is covered) = 0.90

**Fig. 5:**
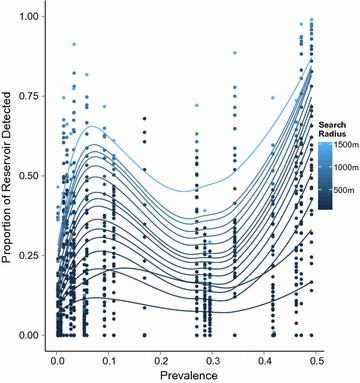
Proportion of reservoir detected vs. prevalence

Increasing search radius or the number of households searched in a RCD re-sample increased the probability of finding a higher proportion of infected individuals. This is necessarily true across both higher and lower prevalence settings. However, even search radii up to 1.5 km found less than 80% of RDT positive individuals in the majority of re-samples (Figs. 4a, 5). These results indicate that RCD, even with such broad search criteria, would still cover fewer than 80% of the existing parasite reservoir over a short period. This also indicates that RCD strategies may take a substantial period to achieve the aim of identifying and reducing an existing parasite reservoir. An example of RCD search radii sweep results are shown in Fig. 4a. Averages over catchment areas showed lower overall proportions of infections detected, than did averages across the whole population. Population aggregate results, shown in red, effectively weight the overall results by catchment area by population and prevalence, whereas averages of catchment level results (shown in blue), present the results as though each catchment was a ‘typical area.’

Meaningful levels of clustering of malaria RDT positive individuals were present in lower prevalence areas. Varying the number of nearby households searched resulted in similar findings, though calibrated on a different scale. With a fixed RCD search radius, lowering the coverage of households within the search area and of individuals within searched households who are tested or treated limited the overall ability of the RCD system to identify infected persons.

### Active fever detection or sweeping treatment-seeking probability

The algorithm allows for individual treatment-seeking probability to be specified. Individuals who reported a history of fever in the previous two weeks were given a probability of seeking treatment. This allowed for the investigation of the impact that failure to seek treatment has on the ability of an RCD system to identify significant portions of the RDT positive reservoir of infected individuals. Increasing the treatment-seeking probability from levels actually observed to much higher values increased the coverage of the RCD system for a given search radius, mainly by increasing the number of index cases generated, this was true in both high and low prevalence areas (Fig. 4b).

**Fig. 6:**
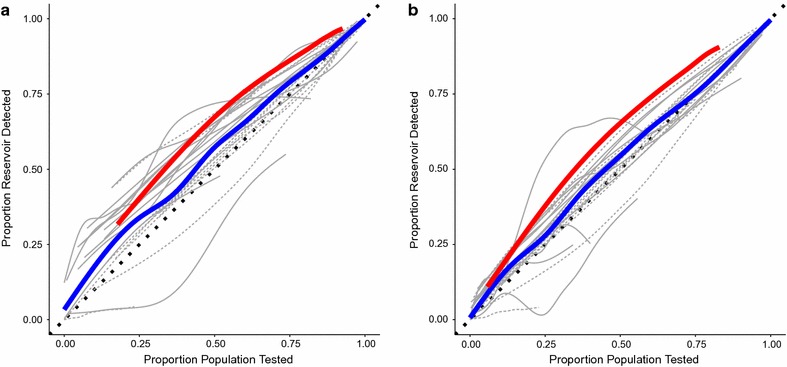
Reservoir detected vs. proportion of population tested by treatment seeking probability. **a** Proportion of reservoir detected vs. proportion of population tested with treatment seeking probability of 0.80; **b** proportion of reservoir detected vs. proportion of population tested with treatment seeking probability of 0.20; *red line* population aggregate, *solid grey lines* catchment areas with prevalence below the median (92/1000), *dashed grey lines* catchment areas with prevalence above the median (92/1000), *blue line* average over catchment areas. Search radius varied from 1–1500 m, field test sensitivity = 0.95, field test specificity = 0.80, P (treatment seeker is tested) = 0.90, P (RCD-selected household is covered) = 0.90, P (individual within RCD-selected household is covered) = 0.90

Treatment-seeking by all febrile cases would result in more efficient identification of malaria infected individuals than random testing in the population (Fig. 6), suggesting that persons testing RDT positive are also clustered around febrile individuals. This may also suggest that improving treatment seeking in the community could in fact improve the efficiency of existing RCD programmes. At high levels of treatment-seeking probability this approach would mimic an active fever screening approach coupled with RCD around each case identified through the active fever screening. The overall efficiency of the approach for identifying infections does not seem to be greatly influenced by the treatment seeking fraction (Fig. 6), despite the fact that the proportion of the reservoir detected is greatly increased (Fig. 4b). Although this approach appears to be efficient in that a higher proportion of all infections are detected compared to the numbers of individuals searched, it would require visiting each household (or large portions thereof) and asking about febrile history prior to conducting the testing and/or treatment. Alternatively, the treatment-seeking fraction might also be increased through expanding the reach of the health system by adding CHWs or additional health centres. Interestingly, the gains are steepest at low ends of treatment-seeking probability, suggesting that the largest gains may come from targeting individuals or areas with the most limited access to care or lowest probability of seeking care with outreach activities or active fever detection.

### Prevalence

Higher proportions of the infectious reservoir were detected in low prevalence areas vs. moderate prevalence areas after controlling search radius and treatment-seeking probability (Fig. 5). Similar or high proportions of infections were also detected in very high prevalence areas. The shape of the curves indicate that there is lower amounts of clustering of malaria infection at moderate prevalence levels, though its possible that this phenomena might be specific for the areas and times studied, or related to the diagnostics used.

### Diagnostic sensitivity and specificity and fDA

**Fig. 7:**
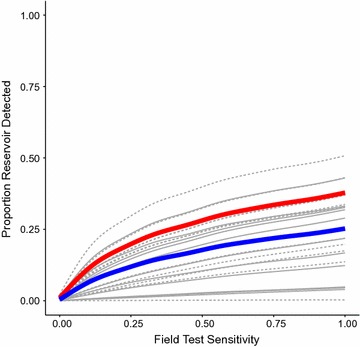
Proportion of reservoir detected vs. field test sensitivity. *Red line* population aggregate, *solid grey lines* catchment areas with prevalence below the median (92/1000), *dashed grey lines* catchment areas with prevalence above the median (92/1000), *blue line* average over catchment areas. Treatment seeking behavior sourced from data, search radius = 100m; field test specificity = 0.80; P (treatment seeker is tested) = 0.90; P (RCD-selected household is covered) = 0.90; P (individual within RCD-selected household is covered) = 0.90

Results for RCD identification of an RDT positive individual are dependent on the sensitivity of the diagnostic used in the RCD search (Fig. 7), as well as the identification of index cases. Under an fDA approach this limitation is removed as all individuals in a given search area would be treated regardless of infection status. Re-samples were conducted by varying the search radius and assuming that the sensitivity of the diagnostic used was perfect. This resulted in increases in the fraction of individuals who tested RDT positive who were identified (and thereby assumed to be treated) relative to RCD where treatment and identification was dependent on a positive diagnostic result across all prevalence settings, however, this increase was modest.

Increasing the diagnostic sensitivity in the field results in a higher proportion of RDT positive individuals identified in the search process though gains are small, even when sensitivity is increased to its maximum levels (Fig. 7). Decreasing the specificity of the diagnostic used in the field will lead to significantly increased numbers of individuals being treated, but does not increase the proportion of the reservoir identified. Increasing the sensitivity of the diagnostic used at the facility increases the proportion of the reservoir identified for a given search radius in low prevalence areas but did not make a substantial change in higher prevalence areas. Decreasing the specificity of the diagnostic used in the facility increased the proportion of the parasite reservoir identified in both low and high prevalence areas, as a consequence of increasing the number of index cases identified and searches conducted. Interstingly, this finding suggests that generating new index cases, or conducting reactive searches around febrile index cases who are not malaria positive, might be an effective reactive strategy. These effects were mitigated at higher search radii as fewer index cases are required to ensure a serach of a large fraction of the popualtion when a large search radius is used. Additionally, in low prevalence areas decreased specificity of the diagnostic used to identify index cases pushed the proportion of identified RDT positives closer to the proportion of the population which was searched, due to increasing the number of RCD searches conducted following false positive results at the health facility.

### Operational results

#### Reactive case detection (sweeping radii or number of households searched)

Increasing search radii or number of households searched in response to an index case led to increases in the number of individuals and households searched (Table 2). However, depending on whether the assumptions of the simulations was that households would be searched only once (a unique search) or whether households might be searched multiple times in response to different index cases which occurred in the same period of time the results varied considerably.

**Table 2:** Operational results

Parameter	Parameter range	Index cases	Unique households searched	Unique individuals searched	Unique individuals treated (fDA)	Parasitological coverage
Treatment seeking probability	0.2–0.3	35 (0–106)	315 (0–1046)	933 (0–2850)	592 (0–1682)	0.12 (0.00–0.40)
	0.7–0.8	107 (1–320)	448 (3–1388)	1459 (3–4822)	1025 (1–3187)	0.22 (0.00–0.71)
	Observed	36 (0–96)	306 (0–969)	915 (0–2651)	598 (0–1609)	0.12 (0–0.42)
Diagnostic sensitivity at clinic	0.2–0.3	60 (0–173)	351 (0–1192)	1087 (0–3439)	731 (0–2125)	0.15 (0.00–0.52)
	0.7–0.8	82 (0–241)	383 (0–1290)	1220 (0–3901)	840 (0–2555)	0.18 (0.00–0.62)
Diagnostic specificity at clinic	0.2–0.3	46 (0–132)	326 (0–1103)	991 (0–3113)	649 (0–1898)	0.13 (0.00–0.47)
	0.7–0.8	96 (1–283)	425 (2–1363)	1367 (1–4536)	950 (0–2899)	0.20 (0.00–0.67)
Diagnostic sensitivity in the field	0.2–0.3	71 (0–205)	367 (0–1248)	1153 (0–3681)	741 (0–2162)	0.12 (0.00–0.40)
	0.7–0.8	71 (0–207)	367 (0–1247)	1155 (0–3691)	845 (0–2575)	0.22 (0.00–0.72)
Diagnostic specificity in the field	0.2–0.3	71 (0–204)	368 (0–1248)	1161 (0–3716)	646 (0–1890)	0.16 (0.00–0.58)
	0.7–0.8	71 (0–205)	367 (0–1247)	1156 (0–3692)	971 (0–2994)	0.16 (0.00–0.58)
Search radius in meters	10–20	71 (0–206)	44 (0–127)	127 (0–351)	69 (0–185)	0.02 (0.00–0.07)
	20–30	71 (0–204)	50 (0–146)	141 (0–402)	76 (0–209)	0.03 (0.00–0.08)
	30–40	71 (0–202)	58 (0–168)	156 (0–446)	84 (0–234)	0.03 (0.00–0.08)
	40–50	72 (0–208)	67 (0–196)	185 (0–541)	101 (0–279)	0.03 (0.00–0.10)
	50–100	71 (0–205)	94 (0–275)	253 (0–719)	139 (0–384)	0.04 (0.00–0.12)
	100–200	71 (0–205)	162 (0–476)	442 (0–1221)	255 (0–698)	0.06 (0.00–0.20)
	500–600	71 (0–206)	362 (0–1194)	1104 (0–3490)	727 (0–2162)	0.15 (0.00–0.51)
	900–1000	71 (0–206)	443 (0–1386)	1420 (0–4776)	982 (0–3095)	0.20 (0.00–0.67)
	1000–1500	71 (0–206)	477 (0–1452)	1565 (0–5351)	1110 (0–3595)	0.23 (0.00–0.75)

All probability parameters were simulated uniformly over the range 0–1. Search radius was simulated uniformly over the range 1–1500. Each row represents the average of all catchment area results meeting the specified parameter range criteria. 80% credible intervals ranging from the 10th to the 90th quantiles of the averaged simulations are included in parentheses

When households were searched uniquely, the number of houses or individuals who would be searched with a RCD approach showed diminishing returns to expansion of the search radius, while the number of household searches which occurred when such searches could occur more than once in a short period (~2 weeks) increased roughly linearly with the increase of radius over a search area.

Differences between the total houses searched in a non-unique search framework and in a unique search framework were considerable, indicating that as search radius was swept to larger values that there was significant overlap in the areas indicated for RCD.

#### Active fever detection or sweeping treatment-seeking probability

Increasing individual treatment-seeking probability led to higher numbers of households and individuals searched (Table 2) when compared to an RCD strategy where observed treatment-seeking levels were used. Similar relationships between unique and repeated searches to those seen using a RCD approach were apparent here. Higher levels of treatment-seeking leading to repeated searches of households in the same search area and diminishing marginal returns to increasing treatment-seeking in terms of how many households would be uniquely searched. These relationships held over both low and high prevalence health facility catchment areas.

#### Diagnostic sensitivity and specificity and fDA

In addition to sensitivity, diagnostic specificity will also affect the performance of a system (Table 2). Reducing the specificity of the diagnostic used for identifying infections in the search process, while holding search radius constant, leads to an increase in the proportion of individuals who were not RDT positive in the MTAT dataset who would be directed to receive treatment in a RCD search. While increasing the sensitivity of the diagnostic results in increasing the number of RDT positive individuals being identified as such in the search process. In the limit for both, the algorithms for RCD approximate those of fDA. These changes do not result in any difference in the number of individuals or households searched relative to RCD with imperfect diagnostics at the field level. However, changes in sensitivity and specificity of the diagnostic used at the facility to identify index cases in the passive-active (RCD) framework do result in changes in both the number of households and individuals searched and in the relationships between the efficiency of the search process.

Increasing the sensitivity of the diagnostic at the facility leads to minimally increased numbers of investigations, households and individuals searched while decreasing the specificity of the diagnostic used at the facility leads to an increase in the number of searches, households and individuals searched for small search radii. Though this effect is mitigated at larger search radii where, overlap between searched households reduces the number of newly searched households for each index case.

## Discussion

RCD and related strategies are used mainly by malaria elimination programmes, and have been deployed widely in Asia though only to a limited extent in Africa. The literature on operational issues, reservoir coverage and programme effectiveness is limited, but has expanded in recent years [5, 6, 11, 16–23]. While some studies identify added value in RCD approaches [18, 22, 23] others identify serious limitations and low parasite reservoir coverage despite large operational efforts [17, 19, 20]. This study used data from a census of the population of part of Southern Province Zambia with parasite detection combined with a novel computer algorithm to estimate the coverage of the parasite reservoir and the operational requirements to conduct RCD and related malaria control interventions. These results show that RCD or active fever detection coupled with RCD and fDA have potential to reach significant portions of the malaria parasite reservoir. However, they also point out some serious challenges with these approaches. These include: (1) that meaningful fractions of the parasite reservoir can be found in a short time period only when large numbers of households and individuals are reached, and (2) using the search radii considered, clustering of RDT positive malaria infected persons led to some efficiencies in parasite detection using an RCD approach but in most of the scenarios considered these were surprisingly small compared to random searches of the same areas, and (3) the RCD process is greatly hampered by low levels of treatment-seeking for fever in health facilities which would be used for identification of index cases in a standard passive-active RCD approach.

While a shift from RCD to an active fever search would mitigate some of the problems with low treatment seeking, it would also require wide population sweeps to identify persons with a history of fever or a current febrile illness as candidate index cases. Such an approach would be operationally challenging and costly to carry out on large scales. Other strategies to change treatment-seeking for febrile illness including the expansion of the health system and behaviour change communication may also be options. For example, fever treatment could be improved by extending testing and treatment services into communities and closer to areas where malaria infections occur. Other methods to increase the number number index cases generated such as decreasing the specificty of criteria for selection of index cases at the facility could also improve reservoir coverage.

The data represents a time when no RCD system was in place in the MTAT areas and when the transmission was much higher than currently in Southern Province. In simulations where reported treatment seeking was used these were based on people attending health facilities, not to local CHWs or other community case management (iCCM) implementers. While the treatment-seeking probability was explored in sensitivity analysis, results of improving treatment-seeking for the average person with fever may not reflect the same patterns as occur with the roll out of CHWs as part of iCCM. Currently RCD systems are being piloted in Zambia and expansion of the health system through the roll out of iCCM has also occurred [24, 25]. These results indicate that RCD in these contexts may indeed capture a significant portion of the parasite reservoir, at least in low transmission areas similar to Southern Province Zambia.

One earlier study used population level summaries from southern Zambia survey data to develop an agent-based transmission model to simulate the population data to which an approach to RCD similar to the one described here was applied [6]. This study takes a different approach by applying re-sampling directly to the census data. Additionally, their study was based on data from four catchment areas while the current study is based on data from 23 catchment areas.

Another earlier study using survey data from southern Zambia used a combination of logistic regression methods, and geographic analysis to estimate the proportion of infected individuals living within a specified radius of a household with a positive malaria RDT result for a person who was positive and sought care [7]. The Searle et al. study, however, had to impute most of the malaria diagnostic, symptomatic and treatment seeking results utilized in the analysis of RCD efficiency because only sample survey datasets and household locations were available to them. In addition, they did not consider the sensitivity and specificity of diagnostics used in the index case identification process, nor did they consider search radii smaller than 500 m or imperfect coverage in the search process. These results indicate that search radii of 500 m or more would result in large operational requirements in southern Zambia and significant amounts of overlap between search areas for different index cases. Additionally, the operational coverage [proportion of households actually searched (of those who should have been searched) and individuals searched with these households] achieved during RCD or related activities is an additionally important parameter central to the functioning of these systems.

This study is limited by several threats to external and internal validity. One major factor is that, though it utilizes census data, which mitigates some limitations of previous studies which had to impute outcomes for a majority of households [7], it still relies on cross sectional data, limiting its’ ability to estimate the coverage of RCD and related interventions when repeated over longer time frames. Only well designed prospective studies of the intervention in context could show this. Secondly, the diagnostics used in the census data collection on which this study is based were RDTs. These tests are known to have limited sensitivity for low density infections [26]. As such, this study may have a limited ability to project the achievement of coverage when such low density infections are included, which may be possible with the development of new highly sensitive RDTs. The direction of bias that arises from this limitation will depend on the nature of clustering of these low density infections in relation to index cases. Unfortunately, it is not possible using the data currently available to be certain whether this bias would result in a lower proportion of all infections being identified with RCD or a higher proportion compared to the results we find using only RDT results. Finally, while these datasets derive from large populations with widely varied parasite prevalence, they come from only one part of Zambia. The results of applying RCD or related interventions to other locations in Africa or the world may be very different, even in terms of coverage achieved, due to variation in human behaviour and settlement patterns and epidemiology of malaria transmission.

This study only estimates the reservoir coverage that might be expected in RCD and related systems over a short period of time (~2 weeks). This neither implies that coverage will remain low over extend periods should RCD or related approaches be sustained, nor that there will be no effect on transmission even at low reservoir coverage (though results for previous studies indicate that even high reservoir coverage in a short period are unlikely to have major effects in this setting [14]). Further simulation, modeling and evaluation work around RCD systems can incorporate these results in parameterization and as guidance on measurement. As such these results should be important to development and implementation of RCD and related approaches in malaria control and elimination programmes.

A number of additional factors will be important to consider in future work in this area. These include generating field trial data and mathematical model based estimates of the effectiveness and efficacy of RCD and related strategies, and identifying the determinants which may affect coverage. Some modelling work on limited data sets from this area of Zambia has already been conducted [6].

Additionally, as this study has shown that the resource requirements of conducting RCD and similar strategies may be significant, a finding consistent with qualitative and quantitative work already undertaken in Zambia [21, 27]. An important next step based on these results will be to measure and attach explicit cost functions to these or similar analyses to determine, under budget constraints, what the optimal parameters of an RCD or fDA intervention (i.e. what search radius) are in a given context.

## Conclusion

RCD, fDA and active fever screening followed by RCD will only yield limited coverage of the RDT positive parasite reservoir over a short time period. Use of reactive strategies as routine tools for an extended period may increase this proportion. Reactive strategies with a fixed radius around an index case detect a higher proportion of the reservoir of infections than similar searches around randomly selected househods, but this effect appears to be greater in areas of low, but not moderate malaria prevalence in southern Zambia. Changes in the detection limit of RDTs could also affect results. The number of individuals who need to be searched, and thus the resource requirements to do so increase rapidly, but approximately linearly with search radius. RCD, if implemented in southern Zambia, would yield higher fractions of the reservoir detected with similar effort if targeted to areas with prevalence less than 10%. Increasing the probability that febrile individuals seek care or the search radius around index cases can both increase the proportion of the reservoir covered by RCD and related strategies, however, both approaches will increase the coverage most quickly when they start from low levels, and neither appears to greatly increase the fraction detected once moderate levels of sensitivity or treatment seeking are reached. The success of an RCD system appears highly dependent on its ability to actually search the houses and individuals that are within target areas for additional malaria infections—programmes implementing these strategies should not neglect the operational aspects of these systems.
